# Alveolar Hemorrhage

**Published:** 1899-09

**Authors:** W. A. White

**Affiliations:** Phelps, N. Y.


ARTICLE VI.

### ALVEOLAR HEMORRHAGE.

DR. W. A. WHITE, PHELPS, N. Y.

In the May and June issues of "Items of Interest," I noticed records of cases of alveolar hemorrhage; one by Dr. H. H. Benjamin, of Bavaria, N. Y., and one by Dr. Charles P. Chupein, of Philadelphia, Pa. Having recently had an unpleasant experience of this kind, and believing it worthy of notice, I send you the facts.

The patient being a gentleman, a prominent business man of North Adams, Mass., aged sixty-one years, during the past two years had been in very feeble health, attributed to a complication of troubles, principaily an aggravated liver disease. He had received treatment by several noted physicians and at various sanitariums, but seemed to be gradually failing. Some six weeks ago he placed himself for treatment under the care of Dr. P——, of this place, and was seemingly improving daily. He wore a full upper denture, and several years ago lost all his teeth back of the second bicuspids on each side of the lower jaw. All of the remaining teeth were broken off to the gum line excepting the cuspid and first bicuspid on the left side; and the second bicuspid on the right side, and these were badly decayed. From these he suffered much, and after several appeals to his attending physician, an appointment was made to have them extracted: At 9.30 a. m., May 26, Mr. D.; in company with his wife and Dr. P. came to my office. Chloroform was administered, and the teeth above mentioned were extracted. Complete anesthesia was produced, and the operation was finished within half an hour, the anesthetic being administered withour any unpleasant symptoms; only the normal bleeding followed. At 10.15 they left the office, the patient apparently being the jolliest one of the three. After arriving at his room, Mr. D. lay down to rest, and dropped off to sleep, at 1 p. m. he had a severe chill, when the doctor was called, and soon gave him relief. Shortly after the physician had left and the rigor had ceased, the hemorrhage began. He had been bleeding profusely for an hour when I was called at 2 p. m., when I found the patient in so weakened a condition that from the first it seemed like a hopeless case. I packed the cavities with pledgets of cotton saturated with phenol sodique and tannin, which was the only hemostatic used. By 3 p. m. the hemorrhage had entirely ceased, and save the exhausted condition of the patient the indications pointed to recovery. So fearful was he that the hemorrhage would return, at his request I remained with him all night. He rested well, and was awakened occasionally to be given his remedies, together with a little beef broth as nourishment. The heart action with the radial pulse was noted at intervals, and was strong, considering the shock the patient had suffered. His condition without change remained the same until 5.30 Saturday a. m., when he grew very restless, and I was hardly able to detect the radial pulse; while the heart gave evidence of weakening. His physician was called, who at once recognized his condition. Dr. H. was summoned in consultation. Both physicians used every means to save the patient, but he died at 12.30, May 27th.

While I am convinced that the debilitated condition of the man was not equal to the operation, I am strongly of the opinion that, had either his physician or myself been called when the hemorrhage first commenced, the result might have been different. It was estimated that he had lost three quarts of blood before aid was summoned.—*Items of Interest.*

